# How decisive is the number of distal arterial anastomoses in coronary bypass surgery?

**DOI:** 10.1186/s13019-020-01384-9

**Published:** 2021-01-07

**Authors:** Olivier J. L. Jegaden, Fadi Farhat, Margaux P. O. Jegaden, Amar O. Hassan, Joel Lapeze, Armand Eker

**Affiliations:** 1 Department of Cardiac Surgery, Mediclinic Middle East, Mediclinic Airport Road Hospital, PO Box 48481, Abu Dhabi, UAE; 2 MBRU University, Dubai, UAE; 3 UCLB University Lyon1, Lyon, France; 4 Department of Cardiac Surgery HCL, Lyon, France; 5 Department of Surgery, Kremlin-Bicetre APHP, Paris, France; 6 Department of Cardiac Surgery, Infirmerie Protestante, Lyon, France; 7 Centre Cardio-thoracic, Monaco, Monaco

**Keywords:** Coronary disease, CABG, Arterial revascularization, Internal thoracic artery, Sequential graft, Gastroepiploic artery

## Abstract

**Background:**

The benefit of arterial revascularization in coronary surgery remains controversial. The incremental value of additional grafts to the left internal thoracic artery (ITA) has been mainly assessed according to the number of arterial grafts, possibly limiting the detection of its actual impact. We analyzed the influence of the number of distal arterial anastomoses (DAA) performed on late mortality in patients having received from one to three arterial grafts.

**Methods:**

Retrospective review of 3685 primary isolated coronary artery bypass grafting (CABG) performed from 1989 to 2014 was conducted with a 13-year mean follow-up. One arterial graft (SITA) was used in 969 patients, two arterial grafts, ITA or gastroepiploic artery (GEA), in 1883 patients (BITA: 1644; SITA+GEA: 239), and three arterial grafts in 833 patients (BITA+GEA). Totally, 795 patients (22%) received one DAA, 1142 patients (31%) two, 1337 patients (36%) three, and 411 patients (11%) four or more. A sub-group analysis was done in the 2104 patients with 3-vessel disease who received at least 2 arterial grafts.

**Results:**

In this series the early mortality was 1.6% and it was not influenced by the surgical technique. Late mortality was significantly influenced by age, gender, heart failure, LV ejection fraction, diabetes status, complete revascularization, number of arterial grafts, number of DAA, both ITA, sequential ITA graft, GEA graft. In multivariable analysis with Cox regression model, the number of DAA was the only technical significant independent prognosis factor of late survival (*p* < 0.0001), predominant over both ITA, complete revascularization and number of arterial grafts. The impact of the number of DAA on survival was found discriminant from 1 to 3; after 3 there was no more additional effect. In 3-vessel disease patients who received at least 2 arterial grafts, the number of DAA remained a significant independent prognosis factor of late survival (*p* < 0.0001).

**Conclusions:**

The number of distal arterial anastomoses is an independent predictor of long-term survival, predominant over the number of arterial grafts and the completeness of the revascularization; higher the number, better the late survival. It is a strong support of the extensive use of arterial grafting in CABG.

## Background

Long term advantages of multi-arterial grafting in patients undergoing coronary bypass surgery (CABG) has been controversial for decades despite mounting evidence, but never strong enough to convince surgeons to change their practice [1–4]. There was a huge expectation from the 10-year multicenter randomized arterial revascularization trial (ART) which finally reported comparable 10-year outcomes of bilateral internal thoracic artery (BITA) grafting relative to single internal thoracic artery (SITA) grafting [5]. Interestingly, almost all studies in this topic were based on the analysis of long-term results according to the number of arterial grafts: SITA, BITA, additional radial artery (RA) graft or gastro-epiploic (GEA) graft [6–9], and not according to the number of arterial anastomoses performed which define the concept of arterial revascularization more accurately. We analyzed our 25-year experience in arterial grafting to test the hypothesis that the number of distal arterial anastomoses (DAA) performed in patients undergoing CABG has an impact in their long-term outcome.

## Methods

### Patients

All patients who underwent isolated CABG procedure in our department that was performed by the same surgeon (OJ) from January 1989 to August 2014 were selected in the study, regardless of the surgical technique used. Exclusion criteria were single vessel coronary disease, emergency, reoperation, associated procedure, and an unstable situation with Troponin rise. We retrospectively analyzed the prospectively collected data from the surgical registry of the department. This was approved by both the local ethical committee and after receiving prior consent from the patient. Finally, 3685 patients were selected: 969 patients received a single arterial graft, 1883 patients received 2 arterial grafts and 833 patients received 3 arterial grafts.

### Surgical technique

Our surgical techniques in CABG were previously reported in studies focused on early postoperative outcome [10], with a unicast configuration according to the grafts used. In single ITA grafting procedure, patients received a single ITA graft (SITA) to the left anterior descending artery (LAD) plus supplemental vein grafts or GEA graft to other coronary arteries. In bilateral ITA grafting procedure, patients received both left and right ITA (LITA and RITA) grafts to the most important coronary arteries on the left side: RITA crossing in front of the aorta to the LAD system and LITA to the circumflex artery (CX) system, with supplemental vein grafts or GEA graft to other coronary arteries. All arterial grafts (LITA, RITA, GEA) were used as in situ grafts preferentially and as composite grafts exceptionally. GEA was used to bypass the right coronary artery (RCA) system exclusively. In SITA and BITA configurations, sequential ITA graft and sequential vein graft were performed according to the coronary lesions and the technical possibilities. The surgical technique was individually chosen in each case according to the state of the patient and the habits of the surgeon; the main concern was no increase in mortality or morbidity. The concept of arterial revascularization with at least 2 arterial grafts was the primary choice as often as possible and it had been performed in 74% of cases, with a yearly rate from 40 to 94%. Eventually vein grafts were most often used in worse cases with heart dilation, severe LV dysfunction, heart failure symptoms, obesity, or when the diameter and the flow of the arterial graft was estimated insufficient. Diabetes status or severe dyslipidemia were never a limitation. CABG was done on-pump with antegrade and retrograde crystalloid cardioplegia. Complete myocardial revascularization was defined as bypass of all significant lesions defined as more than 70% stenosis. All patients received aspirin antiplatelet therapy postoperatively. Postoperative statin and beta-blockers became common practice over the years.

### Definitions and end-point

Early mortality was defined as any death within 30 days of CABG. Late death was defined as death occurring after 30 days from surgery. All causes of mortality was used to assess the long-term outcome. The latest survival status of the patients was obtained in 2019 from the National Institute of Statistics and Economic Studies (INSEE) and a genealogy agency in case of lack of information; the common closing date for follow-up was December 01, 2019. The primary end-point was overall mortality including early death, from any cause, and was analyzed according to the potential risk factors and the surgical configuration. The independent prognosis factors of long-term mortality were established in the all patient population and then they were tested in 3-vessel patients who received at least two arterial grafts to assess their accuracy.

### Statistical analysis

Descriptive statistics for categorical variables are reported as number and percentage; continuous variables are reported as mean ± standard deviation. Continuous variables were compared using Student’t-test and ANOVA; categorical variables were compared using χ^2^ or Fisher’s exact test. Overall survival was estimated using the Kaplan-Meier method and reported as percentage (95% confidence interval). The stratified log rank test was applied to compare the equality of the survival curves. Univariable analyses of predictors of all-cause death were done with binary logistic regression. Multivariable Cox regression analysis was used to identify independent predictors of all-cause death. When a variable was rejected in the model because of linearly dependent to another one, both were introduced separately and alternatively. A 2-tailed *P* value < 0.05 was always considered to indicate statistical significance. All statistical analyses were performed using IBM-SPSS Statistics software version 25.0 (IBM-SPSS Inv, Armonk, NY).

## Results

The data are summarized in Table 1 for all patients and according to the number of arterial grafts used. Multiarterial grafting was performed in younger patients, more in men, less in patients with significant heart failure defined as NYHA class ≥2 and in patients with left ventricular dysfunction and impairment of ejection fraction. Finally, one arterial graft (ITA) was used in 969 patients, two arterial grafts in 1883 patients (BITA: 1644; SITA+GEA: 239), and three arterial grafts in 833 patients (BITA+GEA). In multiarterial groups, complete revascularization was higher, sequential ITA graft and associated GEA were more frequent, and associated vein graft less. Interestingly, in the 2716 patients who received at least two arterial grafts, a vein graft was associated in 22% of cases, mainly as a complement to BITA in alternative to GEA when it was not suitable. Totally, 795 patients had one DAA, 1142 patients had two DAA, 1337 patients had three DAA, and 411 patients had at least four DAA.

**Table 1:** Comparison of preoperative clinical variables and postoperative outcome by patients groups

	All patients (***N*** = 3685)	One arterial graft (***N*** = 969)	Two arterial grafts (***N*** = 1883)	Three arterial grafts (***N*** = 833)	***P*** value
**Age -- year**	64 ± 10	66 ± 10	64 ± 10	61 ± 9	0.001
**Male sex**	3142 (85%)	750 (77%)	1599 (84%)	793 (95%)	0.001
**Heart Failure NYHA ≥ 2**	526 (14%)	293 (30)%	176 (9%)	57 (7%)	0.001
**Diabetes**	590 (16%)	210 (17%)	311 (16%)	69 (8%)	0.004
**3-vessel disease**	2657 (72%)	553 (57%)	1282 (68%)	822 (99%)	0.001
**Left main lesion**	620 (17%)	170 (18%)	352 (19%)	98 (12%)	0.005
**LV ejection fraction %**	59 ± 13	51 ± 16	60 ± 12	61 ± 12	0.001
**Distal Anastomoses**	3 ± 0.8	2.9 ± 0.9	2.9 ± 0.8	3.4 ± 0.6	0.001
**Distal Arterial Anastomoses**	2.4 ± 1	1.2 ± 0.4	2.5 ± 0.6	3.4 ± 0.6	0.001
**DAA = 1**	795 (22%)	795 (82%)	0	0	0.001
**DAA = 2**	1142 (31%)	163 (16.9%)	979 (52%)	0	
**DAA = 3**	1337 (36%)	9 (0.9%)	813 (43%)	515 (62%)	
**DAA ≥ 4**	411 (11%)	2 (0.2%)	91 (5%)	318 (38%)	
**Single ITA**	1208 (33%)	969 (100%)	239 (13%)	0	N/A
**Both ITA**	2477 (67%)	0	1644 (87%)	833 (100%)	N/A
**Sequential ITA**	1377 (37%)	162 (17%)	895 (48%)	320 (38%)	0.001
**Associated GEA**	1072 (29%)	0	239 (13%)	833 (100%)	N/A
**Associated Vein**	1572 (43%)	969 (100%)	599 (32%)	4 (5%)	0.001
**Complete Revascularization**	2284 (62%)	460 (47%)	1130 (60%)	694 (83%)	0.001
**Clamp Time – min.**	51 ± 14	44 ± 12	49 ± 14	59 ± 13	0.01
**CPB time – min.**	68 ± 21	66 ± 17	65 ± 17	76 ± 27	0.01
**1-month Mortality**	59 (1.6%)	21 (2.1%)	27 (1.4%)	11 (1.3%)	0.432
**Mean Follow-up--year**	13.1 ± 7.3	11.5 ± 7.4	12.9 ± 6.9	15.1 ± 7.5	0.001
**Lost of follow-up at 1 year**	112 (3%)	34 (3.5%)	52 (2.7%)	26 (3.1%)	0.321

*NYHA* New York heart association, *LV* Left ventricular, *DAA* Distal arterial anastomoses, *ITA* Internal thoracic artery, *GEA* Gastro-epiploic artery, *min*. Minutes, *CPB* Cardio-pulmonary bypass, *N/A* Non appropriate

### Early results

In this series the early mortality was 1.6% and it was not significantly influenced by the number of arterial grafts (Table 1), the number of ITA used (SITA, 1.9%; BITA, 1.4%; *p* = 0.115) or the number of DAA performed (one DAA, 2.6%; two DAA, 1.8%; three DAA, 0.9%; and at least four DAA, 1.2%, *p* = 0.054). The main postoperative complications are summarized in Table 2; the rate of reoperation for bleeding was significantly higher in multiarterial grafting. The rate of mediastinitis was significantly higher in diabetic patients (1.5% vs 0.4%, *p* < 0.001) and in case of bilateral ITA grafting (0.8% vs 0.1%, *p* = 0.023), to reach 2% in diabetic patients undergoing BITA (*p* = 0.007). There was no difference in early cardiac death according to the graft configuration.

**Table 2:** Postoperative complications and early outcome by patients groups

Complications	All Patients (*N* = 3685)	One arterial graft (*N* = 969)	Two arterial grafts (*N* = 1883)	Three arterial grafts (*N* = 833)	***P*** Value
**Myocardial infarction**	**63 (1.7%)**	**18 (1.9%)**	**24 (1.3%)**	**21 (2.5%)**	**0.064**
**Ventricular arythmia**	**35 (0.9%)**	**12 (1.2%)**	**15 (0.8%)**	**8 (0.9%)**	**0.514**
**Reoperation bleeding**	**51 (1.4%)**	**9 (0.9%)**	**23 (1.2%)**	**19 (2.3%)**	**0.034**
**Low cardiac output**	**31 (0.8%)**	**10 (1.1%)**	**15 (0.8%)**	**6 (0.7%)**	**0.736**
**Stroke**	**13 (0.4%)**	**4 (0.4%)**	**4 (0.2%)**	**5 (0.6%)**	**0.272**
**Mediastinitis**	**21 (0.6%)**	**2 (0.2%)**	**15 (0.8)**	**4 (0.5%)**	**0.129**
**Abdominal event**	**8 (0.2%)**	**1 (0.1%)**	**4 (0.2%)**	**3 (0.4%)**	**0.504**
**Pneumonia**	**21 (0.6%)**	**5 (0.5%)**	**9 (0.5%)**	**7 (0.8%)**	**0.495**
**All complications**	**243 (6.6%)**	**61 (6.3%)**	**109 (5.8%)**	**73 (8.8%)**	**0.014**
**Early mortality**	**59 (1.6%)**	**21 (2.1%)**	**27 (1.4%)**	**11 (1.3%)**	**0.432**
**Cardiac Causes**	**33 (56%)**	**12 (57%)**	**14 (52%)**	**7 (64%)**	**0.794**

### Long-term results

The mean postoperative follow-up was 13.1 ± 7.3 years and 92% complete: 2021 late deaths (56%) occurred (mean delay 11 ± 5 years), 1310 patient (36%) were alive (mean follow-up 17 ± 6 years) and 295 patients (8%) were lost of follow-up, mainly foreign citizens (112 patients during the first postoperative year, and 183 patients after 7.7 ± 4.2 years). Several preoperative and intraoperative variables were identified as significant risk factors of all causes mortality by univariable analysis: age, gender, heart failure, LV ejection fraction, diabetes status, complete revascularization, number of arterial grafts, number of DAA, both ITA, sequential ITA graft, GEA graft (Table 3); however, the mortality was not affected by the 3-vessel disease status and the use of an associated vein.

**Table 3:** Variables influencing mortality by univariable analysis in all patients and in the sub-group of patients with 3-vessel disease and at least two arterial grafts

	All Patients		Sub-group	
Predictor	HR (95% CI)	***P*** Value	HR (95% CI)	***P*** Value
**Preoperative**				
**Age**	1.077 (1.068–1.086)	0.0001	1.070 (1.059–1.080)	0.0001
**Male gender**	0.868 (0.759–0.994)	0.031	1.114 (0.854–1.454)	0.425
**Heart Failure NYHA ≥ 2**	1.122 (1.008–1.249)	0.035	1.546 (1.350–1.771)	0.0001
**Diabetes**	1.302 (1.025–1.656)	0.031	1.314 (1.006–1.717)	0.045
**3-vessel disease**	1.040 (0.902–1.198)	0.591	NA	
**LV ejection fraction**	0.986 (0.980–0.993)	0.0001	0.983 (0.976–0.990)	0.0001
**Intraoperative**				
**Complete revascularization**	0.713 (0.609–0.834)	0.0001	0.730 (0.612–0.871)	0.0001
**Number arterial anastomoses**	0.726 (0.678–0.778)	0.0001	0.838 (0.746–0.941)	0.003
**Number arterial grafts**	0.710 (0.646–0.780)	0.0001	1.044 (0.875–1.244)	0.634
**Both ITA**	0.534 (0.463–0.616)	0.0001	0.784 (0.511–1.202)	0.264
**Sequential ITA**	0.592 (0.518–0.678)	0.0001	0.688 (0.579–0.818)	0.0001
**GEA graft**	0.861 (0.746–0.993)	0.040	1.085 (0.913–1.290)	0.353
**Associated vein graft**	1.031 (0.878–1.211)	0.710	0.877 (0.727–1.059)	0.173

*HR* Hazard ratio, *CI* Confidence interval, *NYHA* New York heart association, *NA* Non appropriate, *LV* Left ventricular, *ITA* Internal thoracic artery, *GEA* Gastroepiploic artery

### Long-term survival

**Fig. 1:**
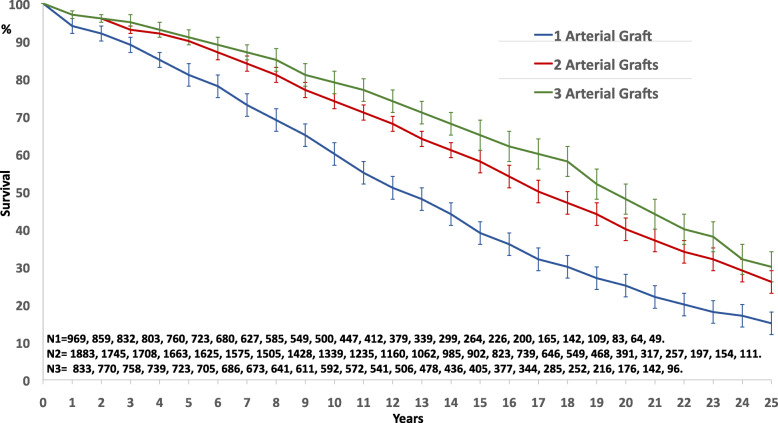
Survival curves by the number of arterial grafts used in all patients (*p* = 0.0001)

**Fig. 2:**
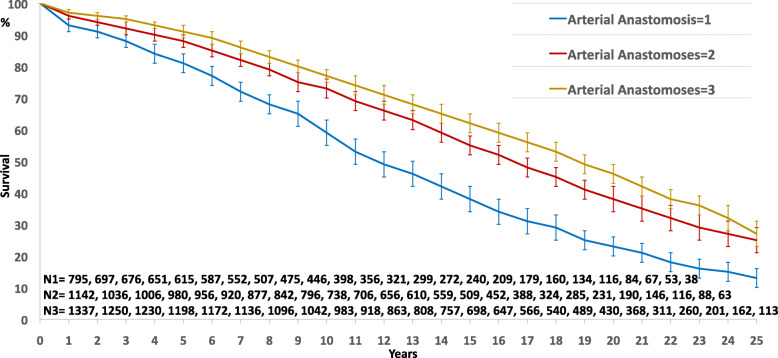
Survival curves by the number of distal arterial anastomoses performed in all patients (*p* = 0.0001)

Long-term survival was significantly better in multiarterial grafting (Fig. 1, *p* = 0.0001). In multivariable analysis with Cox regression model (Chi-square 962,298, df 8, *p* = 0.0001), previous preoperative clinical variables remained independent risk factors of survival. Regarding operative and technical criteria, only the number of DAA was a significant independent prognosis factor of survival; complete revascularization and number of arterial grafts were not identified as independent prognosis factors (Table 4). BITA and GEA graft were found linearly dependent covariates with the number of arterial grafts, and sequential ITA with the number of DAA; these dependent covariates were introduced separately in the Cox model with consistent results: as the number of arterial grafts, BITA and GEA graft were not independent prognosis factor of survival, and sequential ITA was contributing significantly to the influence of the number of DAA on survival (Table 4). The impact of the number of DAA on survival was found significantly discriminant from 1 to 3 (Fig. 2, *p* = 0.0001). After 3 DAA, there is no more additional effect: the 5-, 10-, 15-, 20- and 25-year survival were respectively 91 ± 2, 77 ± 2, 62 ± 3, 47 ± 3, 30 ± 4 in the 1337 patients who had 3 DAA and 91 ± 3, 77 ± 4, 62 ± 5, 46 ± 6, 32 ± 7 in the 411 patients who had at least 4 DAA (*p* = 0.433).

**Table 4:** Multivariable Cox regression analysis of variables influencing survival in all patients and in the sub-group of patients with 3-vessel disease and at least two arterial grafts

	All Patients		Sub-group	
Predictor	HR (95% CI)	***P*** Value	HR (95% CI)	***P*** Value
**Preoperative**				
**Age**	1.076 (1.069–1.082)	0.0001	1.079 (1.071–1.088)	0.0001
**Male gender**	0.836 (0.730–0.958)	0.010	0.870 (0.723–1.048)	0.339
**Heart Failure NYHA ≥ 2**	1.140 (1.074–1.209)	0.0001	1.194 (1.101–1.296)	0.0001
**Diabetes**	1.536 (1.331–1.774)	0.0001	1.634 (1.370–1.949)	0.0001
**LV ejection fraction**	0.981 (0.977–0.985)	0.0001	0.978 (0.973–0.983)	0.0001
**Intraoperative**				
**Complete revascularization**	0.929 (0.840–1.028)	0.154	0.936 (0.822–1.065)	0.314
**Number arterial anastomoses**	0.899 (0.828–0.975)	0.011	0.878 (0.793–0.973)	0.013
**Number arterial grafts**	1.039 (0.924–1.169)	0.522	1.136 (0.965–1.336)	0.126
**Other dependent covariates**				
**Sequential ITA**	0.879 (0.784–0.987)	0.029	0.832 (0.737–0.939)	0.003
**Both ITA**	0.951 (0.818–1.105)	0.512	1.103 (0.824–1.477)	0.510
**GEA Graft**	0.982 (0.819–1.179)	0.849	0.978 (0.850–1.126)	0.759

*HR* Hazard ratio, *CI* Confidence interval, *NYHA* New York heart association, *LV* Left ventricular, *ITA* Internal thoracic artery, *GEA* Gastroepiploic artery

### Sub-group analysis

**Fig. 3:**
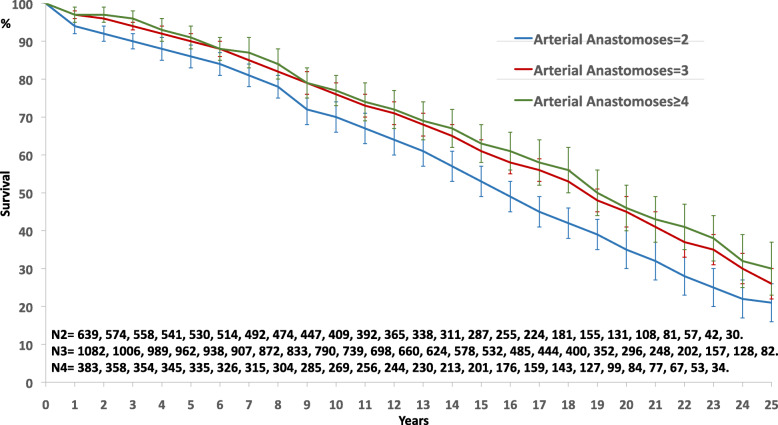
Survival curves by the number of distal arterial anastomoses performed in the sub-group of patients with 3-vessel disease and at least 2 arterial grafts (*p* = 0.001)

A sub-group analysis was done in the 2104 patients with 3-vessel disease who received at least 2 arterial grafts with in mean 2.9 ± 0.7 DAA by patient. They represent 57% of all the population and 79% of the 3-vessel disease patients. In univariable analysis, gender was no more identified as prognosis factors of mortality; complete revascularization, number of DAA and sequential ITA had a significant impact on survival without influence of the grafts used (Table 3). In multivariable analysis with Cox regression model (Chi-square 568,364, df 8, *p* = 0.0001) the number of DAA remained a significant independent prognosis factor of survival, predominant over complete revascularization and number of arterial grafts (Table 4); the impact of the dependent covariates was found unchanged. In this sub-group of patients, the impact of the number of DAA on survival was found significantly discriminant between 2 and 3 (*p* = 0.0001), and low and non-significant (*p* = 0.406) between 3 and at least 4 (Fig. 3).

## Discussion

This study is based on our 25 year-experience of multiarterial grafting as a routine technique. Our strategy was to tailor the operation to the patient according to the coronary network and the estimated operative risk to avoid an increase of early mortality with an obvious bias toward arterial grafting, even if its rate was pretty high with 74% over 25 years. The significant difference in preoperative data illustrate some reasons the patients who had SITA grafting did not get a second or a third arterial graft. Interestingly, despite this significant difference in risk factors, there was no difference in early mortality after CABG according to the arterial graft configuration, confirming a posteriori the efficiency of the strategy of revascularization we adopted. However, our results have confirmed the higher risk of mediastinitis in diabetic patients and in bilateral ITA grafting.

The primary end-point of this study was focused on the overall mortality with a mean postoperative follow-up of 13 years. Univariable and multivariable analyses have confirmed the traditional prognosis factors of mortality and survival after CABG. Regarding the arterial grafting configuration, only the number of DAA was identified as an independent prognosis factor of survival, predominant over complete revascularization, both ITA grafting and number of arterial grafts. Because of the heterogeneity of the population, a specific sub-group of patients including only 3-vessel disease patients who received at least two arterial grafts ITA or GEA, was studied: in this more homogeneous group the number of DAA was also identified as an independent prognosis factor of survival. The influence of sequential ITA has appeared significantly predominant, over the graft distribution; which is consistent because in all ITA configuration, sequential ITA provides an one added distal arterial anastomosis.

The incremental value of additional grafts to LITA has been mainly assessed according to the number of arterial grafts used, possibly limiting the detection of its actual benefit. In ART study [5], 22% of the patients who had been randomly assigned to the SITA group also received a second arterial graft in the form of a radial arterial graft; when data were analyzed according to the number of arterial anastomoses received, two or more as compared to single, there appeared to be a meaningful difference in mortality in favor of multiple arterial grafts [9]. Yanagawa B [11]. has reported a meta-analysis showing that total arterial revascularization may improve long-term survival compared to conventional CABG by 15–20% even when compared to two arterial grafts, suggesting a discriminant impact of the number of distal arterial anastomoses between 2 and more than 2. Buxton B [12]. has reported a large multicenter study showing a 27% difference in survival at 15 years between SITA group who received 1 DAA and total arterial revascularization group who received in mean 3.4 DAA; interestingly this difference is close to what we have observed between patients who underwent 1 DAA and patients who underwent at least 3 DAA with respectively a 38 ± 4% and 62 ± 2% 15-year survival. Even more, Kieser T [13]. have suggested that a strategy of multiple ITA grafts may balance survival between complete and incomplete revascularization that is one of our results as well.

This study confirms that almost all patients in whom multiple arterial grafting is technically feasible and can be safely performed are likely to derive a significant long-term survival benefit [6, 8]. Our technical strategy was confirmed regarding the efficacy of the in situ RITA for LAD grafting [14]. However, there was a predominance of male gender in multiarterial grafting in our series that was related to frequent anatomic limitations in females: a shorter length of in situ RITA to reach LAD or a smaller ITA’s size to perform sequential grafting. A composite ITA graft is probably a more efficient ITA configuration for multiarterial grafting in female. The impact of a third arterial graft in addition to the bilateral mammary arteries has not been clearly defined and remained controversial [15, 16]. We have chosen to associate the GEA graft to unilateral or bilateral ITA graft to obtain an unicast configuration of arterial grafting and to privilege in situ grafting. However, GEA use is not very popular despite the good outcome observed in several studies [17, 18]. Ruttmann E [19]. for RADIAL investigators has reported better survival and outcome regarding patency and the need for target-vessel revascularization of the radial artery graft in comparison with saphenous vein graft; we have had no experience with radial artery grafting and it was never used in this series because we have privileged in situ grafts. We did not test if the impact of ITA anastomosis and GEA anastomosis could be different, first because such evaluation is not appropriate without concomitant study of the patency and second because the target vessels were definitively different; however the contribution of sequential ITA graft was found determinant, underlying the benefit of additional bypass targets revascularized with an ITA.

Despite its inherent limitation, this study has demonstrated that higher the number of distal arterial anastomoses, better the long-term survival. The better patency of arterial grafts have been involved to explain their positive impact; the hypothesis that arterial grafts has a strong protective effect against progression of native coronary artery disease in previously grafted vessels has been reported [20] and multiple arterial grafting may improve long-term survival by preventing progression of atherosclerosis in the native coronary vessels. More recently, Bakaeen F [21]. showed that bypassing multiple targets to maximize myocardium mass supplied by ITAs may improve long-term survival; it could explain our finding, that number of distal arterial anastomoses is finally more accurate and discriminant than the number of the arterial grafts to detect the impact on survival of a multi-arterial grafting strategy.

### Study limitations

The present study has several limitations, inherent to its design and objectives. This is a retrospective observational nonrandomized study based on a 25-year single center, single surgeon and single technical configuration operative experience. Only patients operated on after 1989 were included with a yearly rate of coronary revascularization with at least 2 arterial grafts higher than 40% to exclude the learning curve. Only solid preoperative characteristic and documented for all patients were integrated in the risk factors analysis: for example Euroscore or STS score were missing before 2000, obesity status was not defined properly according to BMI, and they were not included. Nevertheless, the preoperative characteristics included are recognized as the main risk factors of CABG, defining well our CABG population and were completed for all patients. The operative parameters were more exhaustive and precise, allowing a robust analysis of the operative configuration. Only long-term survival and all-cause mortality were defined as primary end-point of the study. It was not the intent of the study to report on other major adverse events as myocardial infarction, repeat revascularization, cause of death, or on graft patency, and the collection of such information was not realistic in this study of over 30 years. Cox regression was used to identify independent predictors of late outcomes and it was estimated strong enough to integrate the bias associated with extensive arterial revascularization. It was not appropriate to differentiate the impact between second ITA, sequential ITA graft and GEA graft because their respective use was not alternative.

## Conclusions

According to the results of this study, the number of distal arterial anastomoses is an independent predictor of late mortality and long-term survival after CABG, predominant on the number of arterial graft used and the completeness of the myocardial revascularization. This criteria could allow a quantitative analysis of arterial revascularization, better reflecting the myocardial mass perfused by arterial grafts than the number of arterial conduits. It is a strong support of the extensive use of arterial graft in CABG. Our finding that higher the number of distal arterial anastomoses, better the long-term survival has to be confirmed in further studies.

## Data Availability

The data sets used and analyzed during the current study are available from the corresponding author.

## Abbreviations

CABG: Coronary artery bypass grafting
ITA: Internal thoracic artery
SITA: Single internal thoracic artery
BITA: Bilateral internal thoracic artery
RA: Radial artery
GEA: Gastroepiploic artery
DAA: Distal arterial anastomoses
LITA: Left internal thoracic artery
RITA: Right internal thoracic artery
LAD: Left anterior descending artery
CX: Circumflex artery
RCA: Right coronary artery
LV: Left ventricle
INSEE: National institute of statistics and economic studies
